# Atypical ulcerative cutaneous tuberculosis revealing disseminated mycobacterial infection: case report with diagnostic and therapeutic challenges

**DOI:** 10.1186/s40249-025-01377-7

**Published:** 2025-11-06

**Authors:** Serena Vita, Andrea Mariano, Francesca Faraglia, Daniele Colombo, Alessandra Scarabello, Gina Gualano, Claudia Palazzolo, Alberta Villanacci, Antonia Maria Olivieri, Franca Del Nonno, Enrico Mirante, Stefania Ianniello, Alessandra D’Abramo, Emanuele Nicastri

**Affiliations:** 1 National Institute for Infectious Diseases, “Lazzaro Spallanzani” - IRCCS, Rome, Italy; 2 Emergency Department Sant’Eugenio Hospital, Rome, Italy

**Keywords:** Cutaneus tuberculosis, Extrapolmunary tuberculosis, Atypical tuberculosis, Ulcerative lesion

## Abstract

**Background:**

Cutaneous tuberculosis (CTB) is an unusual manifestation of extrapulmonary tuberculosis, accounting for only 1.0%–1.5% of cases. It presents with a wide range of clinical morphologies, often mimicking other dermatoses such as fungal infections, leprosy, or sarcoidosis. Among its different variants, the ulcerative form is particularly rare and clinically deceptive. Reporting rare presentation is important to raise awareness among physicians, as early recognition and prompt treatment are essential to prevent complications such as scarring, contractures, or malignant transformation.

**Case presentation:**

We report the case of a 24-year-old Malian male admitted to the National Institute for Infectious Diseases Lazzaro Spallanzani. The patient presented with a 4-month history of ulcerative skin lesions on the chest, neck, and left leg, accompanied by systemic symptoms including asthenia, cachexia, and generalized lymphadenopathy. Imaging revealed extensive bilateral psoas abscesses, vertebral involvement consistent with spondylodiscitis, and signs of empyema necessitans. Polymerase chain reaction (PCR) testing of drained abscess fluid confirmed *Mycobacterium tuberculosis* complex. Skin biopsy histology and PCR further supported the diagnosis of CTB. The patient was treated with standard anti-tuberculosis therapy (isoniazid, rifampicin, ethambutol, pyrazinamide) alongside broad-spectrum antibiotics. After 30 days, partial improvement of skin lesions was observed, although complete resolution was not achieved after 8 months of follow-up.

**Conclusions:**

This case highlights the diagnostic challenge and chronicity of CTB, particularly in the ulcerative presentation. The patient developed disseminated tuberculosis with cutaneous involvement without any recent travel or known tuberculosis exposure, and the probable etiology is latent reactivation. There should be a high index of suspicion for CTB in patients presenting with indolent, atypical skin lesions, particularly those from an endemic region. Early diagnosis and prolonged therapy are crucial to avoid long-term sequelae.

**Graphical Abstract:**

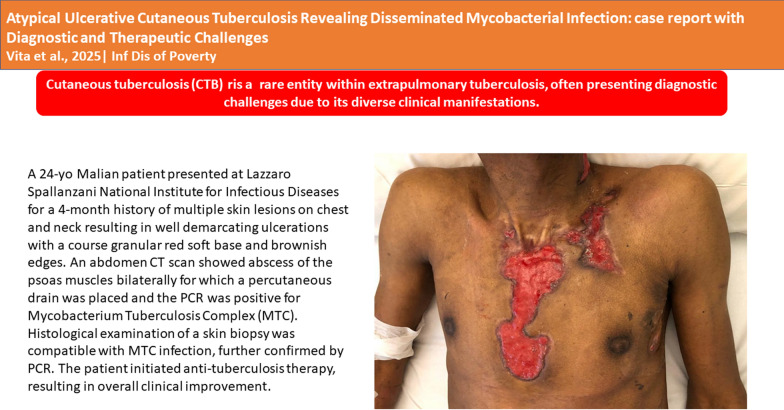

## Background

Tuberculosis (TB) infection affect 10.6 million persons annually [1]. Cutaneous tuberculosis (CTB) is a rare infection that represents 1% to 1.5% of extrapulmonary TB [2, 3]. First described in the 19th century, CTB remains uncommon, with its physiological mechanisms still not fully understood [4]. Like most other forms of TB, CTB is primarily caused by *Mycobacterium tuberculosis*, though *M. bovis* or the Bacillus Calmette-Guérin (BCG) vaccine can also be responsible. The clinical presentation of CTB is highly complex, exhibiting distinct morphological and histological signatures in its various forms. Lesions can mimic several other dermatological conditions appearing as verrucous plaques, suppurative nodules, inflammatory papules, chronic ulcers, and more, making diagnosis particularly challenging [5–9].

The primary manifestations of CTB consist of scrofuloderma, which results from the skin extension of tuberculous lymphadenitis, and lupus vulgaris, characterized by apple-jelly nodules, atrophic scarring, and tuberculosis verrucosa cutis (TVC), which occurs due to direct skin contact with *M. tuberculosis*. Additionally, tuberculids represent hypersensitivity reactions to TB foci [8, 9]. These variants often pose diagnostic challenges due to their varied clinical morphology and their resemblance to other dermatological disorders, such as fungal infections, leprosy, and sarcoidosis. Proper dermatological diagnosis, alongside accurate clinical recognition, is essential for timely and effective treatment [9].

We present a rare CTB case in a young Malian patient admitted at National Institute for Infectious Diseases Lazzaro Spallanzani in Rome on June 2024.

## Case presentation

### Patient information

A 24-year-old Malian patient admitted at National Institute for Infectious Diseases Lazzaro Spallanzani National Institute for Infectious Diseases in Rome on June 28, 2024. In the past six years, he had been living in Italy with no travel abroad. His past medical history was unremarkable.

### Clinical findings

**Fig. 1:**
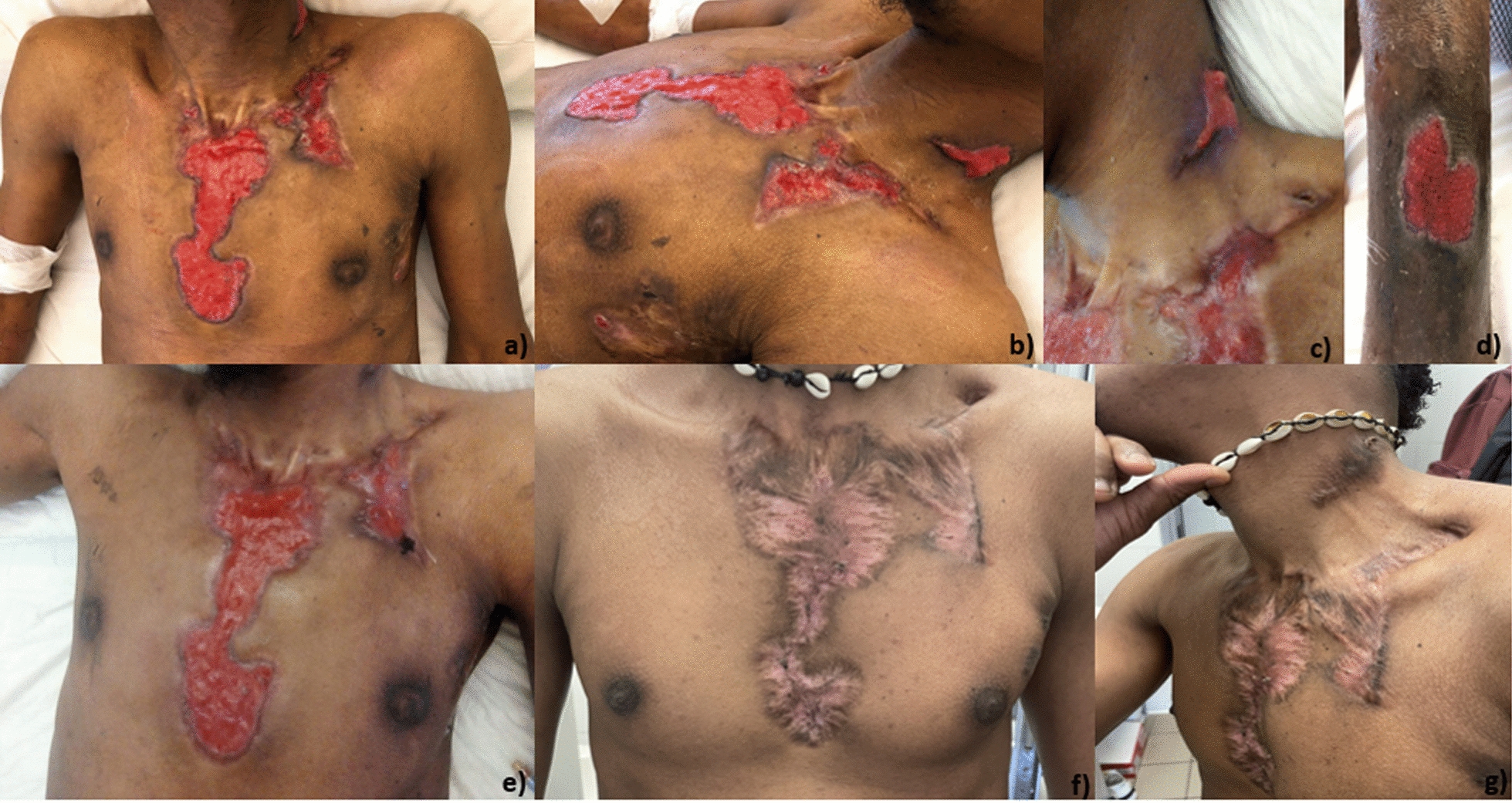
Cutaneous ulcerative lesions at hospital admission on **a** chest **b** chest and neck, **c** neck, **d** leg. In **e** the skin lesions after 30 days anti Tuberculosis (TB) treatment and **f** and **g** after 8 months of anti TB treatment

He reported a 4-months history of multiple skin lesions on chest and neck. These lesions had progressed to well-defined ulcerations with a granular erythematous soft base and brownish edges. A similar lesion was present on the left leg (Fig. 1a–d). He was afebrile, asthenic and cachectic with nummular cutaneous dyschromia affecting both upper and lower extremities. Bilateral, mobile, and mildly tender lymphadenopathies were detected in the laterocervical, axillary, and inguinal regions. His body mass index (BMI) was 17, consistent with underweight status.

### Laboratory and imaging studies

**Fig. 2:**
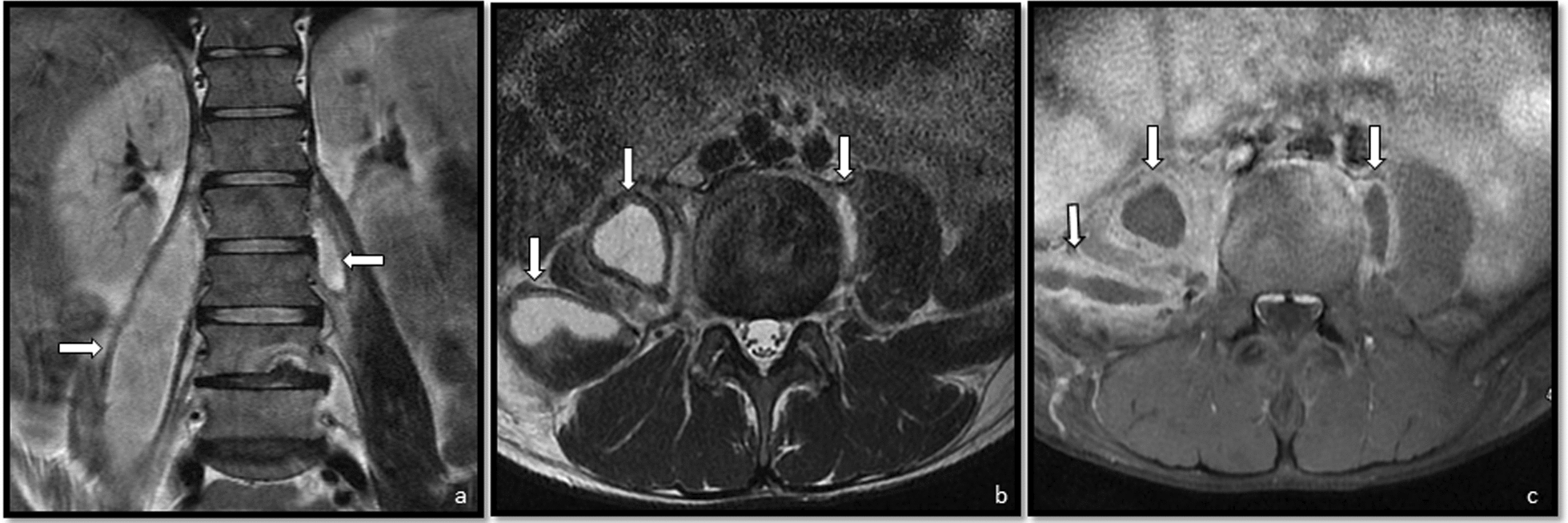
Fluid collection, hyperintense on T2-weighted images (**a**, **b**) and hypointense on T1-weighted images (**c**), consistent with abscess: on the right, located along the iliopsoas muscle, originating at the L1–L2 level and extending caudally to S2; on the left, involving the psoas major muscle between L2 and L3.

Laboratory tests revealed anemia (Hb 78 g/L), normal white cell and platelet counts, normal transaminase levels and elevated C-reactive protein (115 mg/L, reference range <1 mg/L). A contrast-enhanced abdominal computed tomography (CT scan) revealed extensive bilateral psoas abscesses (up to 19 × 7.5 cm on the right), with right-sided extension into the iliacus and abdominal oblique muscles and involvement of the inguinal region. Associated findings included erosive L3 vertebral involvement suggestive of spondylodiscitis, splenomegaly with possible microabscesses, hepatomegaly, multiple enlarged lymph nodes (para-aortic, interaortocaval, iliac, and axillary—some with liquefactive changes), and a right diaphragmatic pleural effusion likely representing empyema necessitans (Fig. 2).

### Microbiological and histological diagnosis

**Fig. 3:**
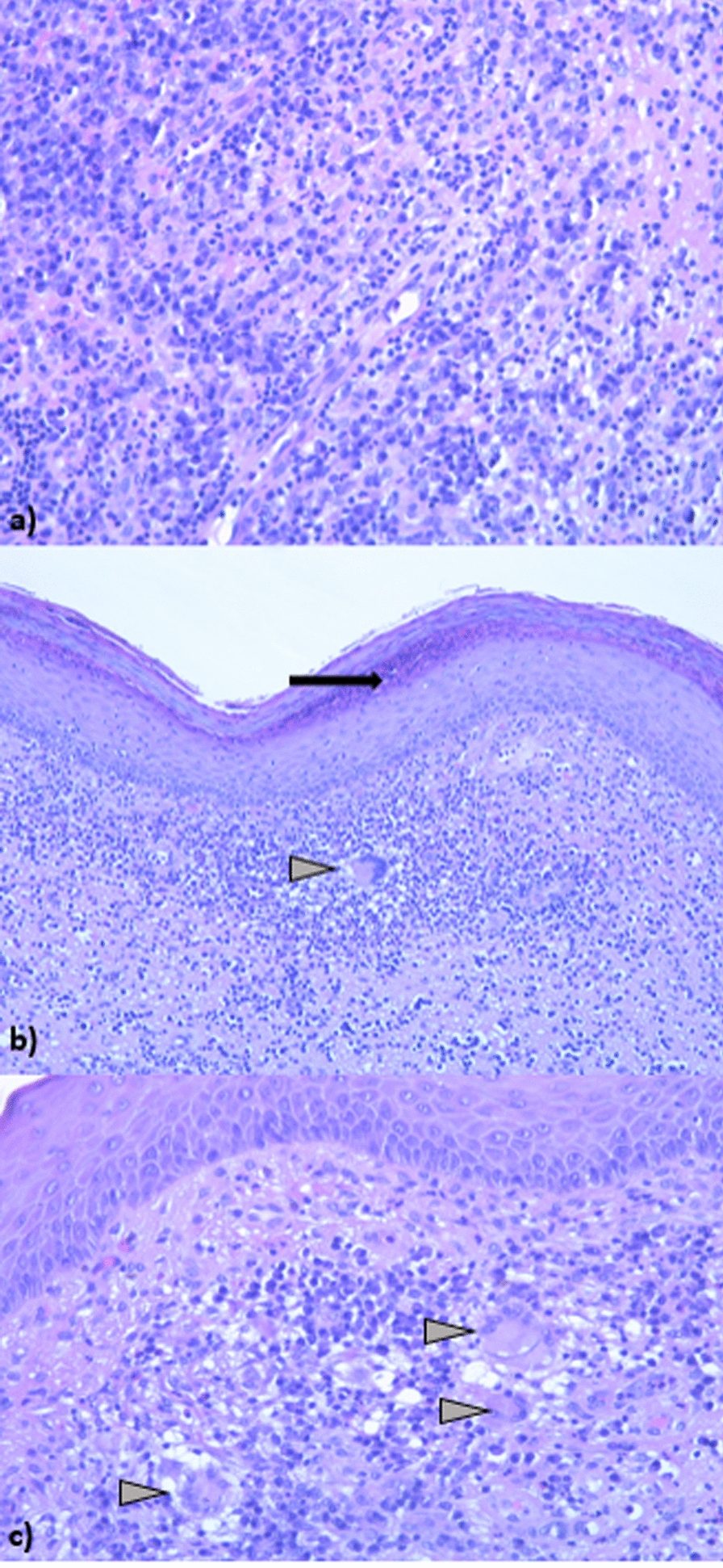
Histology shows a parakeratotic hyperkeratosis with the presence of neutrophil granulocytes in micro-axis level of the stratum corneum; chronic inflammatory infiltrate in the superficial and deep dermis consisting of lymphocytes, plasma cells, neutrophil granulocytes, Langhans cells. **a** Chronic inflammation with many plasma cells in the superficial and deep dermis. Several granulocytes are seen. **b** Skin with parakeratotic hyperkeratosis showing neutrophilic granulocytes (arrow) in the stratum corneum. Langhans giant cell in the superficial dermis (arrowhead). **c** Superficial dermis with Langhans giant cells (arrows)

Percutaneous drainage of the abscess was performed with detection of *M. tuberculosis* complex (MTC) by polymerase chain reaction (PCR) in the drainage fluid, and culture further confirmed the presence of MTC, demonstrating susceptibility to both isoniazid and rifampicin. Histological examination of a skin biopsy was compatible with MTC infection and further confirmed by PCR (Fig. 3). To exclude alternative diagnoses, several investigations were performed. Serologic tests for Hepatitis B and C viruses and HIV were negative. Serology and blood PCR for *Leishmania* were also negative. Culture of the drainage fluid for common bacteria yielded no growth, while PCR assays for non-tuberculous mycobacteria and for *M. leprae* were negative. Moreover, histological examination revealed granulomatous inflammation with caseous necrosis, a pattern strongly suggestive of TB and excluded alternative infectious or non-infectious granulomatous diseases, including sarcoidosis.

### Treatment and outcome

Initially, before the diagnosis of TB was established, the patient was started on broad-spectrum intravenous antibiotics—piperacillin/tazobactam 4.5 g every 8 hours plus daptomycin 500 mg daily—because the clinical presentation suggested a severe bacterial infection, and the etiology was not yet clear. Once the diagnosis of MTC infection was confirmed by PCR (Fig. 3), standard oral antituberculosis therapy was initiated, consisting of isoniazid 300 mg, ethambutol 800 mg, rifampicin 600 mg, and pyrazinamide 1000 mg. After 30 days of anti-TB therapy, a clear improvement in the skin lesions was observed (Fig. 1e). However, complete resolution was not achieved even at eight months of follow-up (Fig. 1f and g), consistent with the prolonged course typically seen in extrapulmonary forms of TB.

## Discussion and conclusions

CTB remains an exceedingly rare entity within extrapulmonary TB, often presenting diagnostic challenges due to its diverse clinical manifestations [5–9]. As seen in this case, the delayed recognition was primarily attributed to the atypical presentation, which deviated from more commonly recognized forms such as scrofuloderma, lupus vulgaris, and TVC. In particular, the ulcerative lesions in our patient, are particularly uncommon, accounting for only 2.6% of cases in an epidemiological study [9]. When compared with larger case series, the rarity of our presentation becomes even more evident. In a retrospective study from Dakar including 151 patients, scrofuloderma (84.7%) and gumma (11.2%) were the predominant clinical forms, whereas ulcerative variants were not reported as a distinct entity [7]. Similarly, in a 20-year Indian series of 280 cases, lupus vulgaris (55%) and scrofuloderma (26.8%) were most frequent, while ulcerative CTB was not described as a common pattern [6]. Furthermore, in both studies, systemic involvement was mainly pulmonary or osteoarticular, whereas the combination of ulcerative skin lesions with extensive psoas abscesses, lymphadenopathy, and vertebral destruction—as seen in our patient—appears uncommon. This case therefore illustrates how CTB, though classically localized, can also manifest as part of a multi-organ disease process. This underscore the need of heightened clinical suspicion for CTB whenever dermatological lesions are atypical, persistent and reefractory to conventional therapies.

Despite the patient having no recent travel history and a relatively unremarkable past medical record, the disease could have originated from either endogenous reactivation of latent TB or recent exposure to MTB in Italy. This aspect reinforces the relevance of CTB not only in endemic countries but also in non-endemic settings, where global migration and latent TB continue to shape the epidemiological landscape.

Although the patient showed clear clinical improvement during the first weeks of specific therapy, complete resolution of the skin lesions was not achieved at the eight-month follow-up. This reflects the chronic and protracted course of CTB, particularly in extrapulmonary forms, where treatment often needs to be extended up to 12 months. In some cases, adjunctive interventions, including surgical debridement, may also be necessary to achieve full resolution. In conclusion, this case highlights three key messages for clinician: the first is that CTB must be considered in the differential diagnosis of chronic, atypical, or non-healing skin lesions, regardless of geographic origin due to global mobility and latent infections. The second is the importance of early recognition and initiation of specific therapy that are essential to avoid systemic dissemination and severe sequelae such as deformities, scarring, or malignant transformation including carcinomas and sarcomas. The last the chronicity of CTB requires long-term follow-up and therapy and, in selected cases, a multidisciplinary approach combining medical and surgical management. Improving clinicians’ familiarity with the varied presentations of CTB is crucial for achieving diagnosis and better patient outcome.

## Data Availability

All data generated or analyzed relating to this study are presented within this published article.

## Abbreviations

CTB: Cutaneus tuberculosis
MTC: *Mycobacterium tuberculosis* complex
PCR: Polymerase chain reaction
TB: Tuberculosis
TVC: Tuberculosis verruca cutis
BCG: Bacillus Calmette-Guérin
BMI: Body mass index
